# Different dynamics of soluble inflammatory mediators after clearance of respiratory SARS-CoV-2 versus blood-borne hepatitis C virus infections

**DOI:** 10.1038/s41598-024-79909-8

**Published:** 2024-11-22

**Authors:** Antonia Zeuzem, Saumya Dileep Kumar, Carlos Oltmanns, Moana Witte, Jasmin Mischke, Nora Drick, Jan Fuge, Isabell Pink, Jan Tauwaldt, Jennifer Debarry, Thomas Illig, Heiner Wedemeyer, Benjamin Maasoumy, Yang Li, Anke R. M. Kraft, Markus Cornberg

**Affiliations:** 1 Department of Gastroenterology, Hepatology, Infectious Diseases and Endocrinology, Hannover Medical School (MHH) OE 6810, Carl-Neuberg-Straße 1, 30625 Hannover, Germany; 2 Centre for Individualised Infection Medicine (CiiM), A Joint Venture Between Helmholtz-Centre for Infection Research and Hannover Medical School, Feodor-Lynen-Straße 11, 30625 Hannover, Germany; 3 German Center for Infection Research (DZIF), Partner Site Hannover-Braunschweig, Hannover, Germany; 4 TWINCORE, Centre of Experimental and Clinical Infection Research, A Joint Venture Between Helmholtz-Centre for Infection Research and Hannover Medical School, Feodor-Lynen-Straße 7, 30625 Hannover, Germany; 5 Cluster of Excellence RESIST (EXC 2155), Hannover Medical School, Carl-Neuberg-Straße 1, 30625 Hannover, Germany; 6 Biomedical Research in Endstage and Obstructive Lung Disease Hannover (BREATH), German Center for Lung Research (DZL), Hannover, Germany; 7 Department of Respiratory Medicine and Infectious Diseases, Hannover Medical School (MHH), Hannover, Germany; 8 Hannover Unified Biobank (HUB), Hannover Medical School (MHH), Hannover, Germany; 9 Department of Internal Medicine and Radboud Institute for Molecular Life Sciences, Radboud University Medical Center, Nijmegen, The Netherlands

**Keywords:** SARS-CoV-2 infection, COVID-19, Long-COVID, Hepatitis C virus, Proteomics, Direct-acting antiviral, Sustained virological response, Cirrhosis, Inflammation, Cytokines, Chemokines, Immune mediators, Immunology, Chemokines, Cytokines, Imaging the immune system, Infection, Infectious diseases, Inflammation

## Abstract

Viral infections can be acute or chronic, with the immune system pivotal in immunopathogenesis. The potential reversibility of inflammation post-viral elimination is of current interest. This study compares the dynamics of soluble inflammatory mediators (SIM) during and after respiratory infections with SARS-CoV-2 and blood-borne acute and chronic hepatitis C virus (HCV) infections. The study included patients with acute HCV (n = 29), chronic HCV (n = 54), and SARS-CoV-2 (n = 39 longitudinal, n = 103 cross-sectional), along with 30 healthy controls. Blood samples were collected at baseline, end of treatment/infection, and during follow-up (up to 9 months). SIMs were quantified using the HD-SP-X Imaging and Analysis System™. At baseline, SIM profiles in acute SARS-CoV-2 and HCV infections were significantly elevated compared with controls. During follow-up, SIM decline was less pronounced in acute and chronic HCV infections after successful therapy than in SARS-CoV-2 infections. Most SIM in the SARS-CoV-2 cohort normalized within 3 months. In chronic HCV, SIM were higher in cirrhotic than noncirrhotic patients post-HCV elimination. Dynamics of SIM after viral elimination vary between blood-borne acute and chronic HCV infections and respiratory SARS-CoV-2 infections. Immunological imprints 3–9 months after HCV elimination appear more pronounced than after SARS-CoV-2 infection.

## Introduction

Viral infections exert a profound influence on the immune system and are capable of inducing immune signatures that persist beyond the acute phase of infection and potentially exert a significant impact on the clinical course^1^. The concept of long-lasting effects of infections on the inflammatory milieu is increasingly being explored, highlighted in particular by the emergence of persistent sequelae following severe acute respiratory syndrome coronavirus 2 (SARS-CoV-2) infections^2,3^, defined as "long-COVID.", which may affect more than 65 million people worldwide^2,4^.

The global spread of SARS-CoV-2 after its initial emergence in December 2019 has shown a spectrum of clinical manifestations ranging from mild flu-like symptoms to severe acute respiratory distress syndrome (ARDS) and fatal outcome^5^. SARS-CoV-2 infections trigger an increase in immune mediators, centering on cytokines and chemokines, which can result in an overshooting reaction, often referred to as “cytokine storm”^6,7^. Long-term effects of SARS-CoV-2 infection often manifest as persistent symptoms of neurocognitive effects, fatigue and dyspnea. The development of Long-COVID has been attributed to tissue impairment, persistent antigen reservoirs, vascular endothelial dysfunction, and autoinflammatory responses which can be determined for example by abnormal concentrations of soluble inflammatory mediators (SIM)^2,3^. This particularly correlates with elevated cytokine levels, which have been shown to be closely associated with systemic inflammation and neurological deficits^2,8^.

However, the magnitude of viral effects on the immune system extends beyond SARS-CoV-2 and, in principle, can occur after multiple infections. Previously, chronic fatigue syndrome (ME/CFS) has been linked to infections or “infectious-like” illness^9^. A particular good example for this is in hepatitis C virus (HCV), which has proven to be a potent catalyst for profound changes in the inflammatory landscape. For example, HCV induces type I interferon responses^10^, which are not necessarily reversible after HCV eradication^11^. HCV infections occur as either acute or chronic episodes, the latter defined by viral persistence of more than six months. Although acute HCV infections may resolve spontaneously within this period, a significant number (50% to 85%) progress into a chronic condition. Chronic hepatitis C can progress to cirrhosis and is associated with an increased risk of hepatocellular carcinoma (HCC)^12^. A significant number of HCV patients also present with various extrahepatic manifestations, such as fatigue or cognitive impairment^13^, which are similar to symptoms observed in individuals with long-term COVID and may persist after HCV clearance^14,15^. It has also been shown that SIM such as CXCL-10 or IFNα are associated with neurocognitive effects and fatigue^16^.

Despite different courses, one being a respiratory infection with acute resolution and the other a potentially chronic blood-borne infection, both SARS-CoV-2 and HCV infections converge in their ability to cause lasting clinical impairment after viral resolution. The profiles and dynamics of inflammatory markers, such as proinflammatory cytokines and chemokines, have been extensively studied and can provide insights into immune status during or after infections^11,17–22^. Therefore, studying the patterns and impacts of these markers could help identify individuals at risk of long-term impairments post-viral elimination. This study aims to explore the dynamics of soluble inflammatory mediators in the contexts of SARS-CoV-2 and both acute and chronic HCV infections, to uncover common and distinct immunologic footprints that shape post-viral immune responses.

## Material and methods

### Study participants and design

Individuals with HCV monoinfection (HIV and HBV co-infections were excluded) were classified into three cohorts (Table 1). The baselines of each HCV cohorts are defined as the day of therapy start with DAA, with the first follow-up being end of treatment (week 6–8).

**Table 1:** Hepatitis C virus cohorts.

Baseline characteristics	Acute HCV	Chronic HCV non-cirrhosis	Chronic HCV cirrhosis
Number of patients	29	23	31
Sex (f/m)	6/23	12/11	0/31
Median age (min–max)	45 (23–63)	56 (18–82)	56 (41/72)
Median BMI (min–max)	23.3 (19–27.7)	25 (17.6–37)	28.1 (25.5–36.2)
Median quantitative HCV RNA	42,700 (1,470–8,000,000)	2,569,350 (55,000–11,000,000)	1,292,575 (235–7,400,000)
Median thrombocytes in Tsd/µl (min–max)	232 (167–362)	209 (101–338)	104 (42–224)
Median hemoglobin in g/dL (min–max)	15.0 (13.6–16.1)	14.7 (10.3–16.8)	14.3 (10.3–16.9)
Median AST in U/L (min–max)		46 (27–139)	111 (15–319)
Median ALT in U/L (min–max)	222 (38–1494)	62 (16–183)	101 (15–284)
Median albumin in g/L (min–max)		42 (35–48)	36 (26–44)
Median leukocytes in Tsd/µL (min–max)		6.5 (4–9.9)	5.2 (2.6–7.5)

Cohort 1 includes acute HCV patients who had participated in prospective clinical trials, with a collective cohort of 29 participants recruited from the German national HepNet acute HCV trials IV (aHCV-IV) and V (aHCV-V)^23,24^. In the aHCV-IV study, sampling occurred at DAA therapy start (defined as baseline) and at the end of treatment (week 6), as well as at follow-up 3 and 6 months after the end of therapy. In the aHCV-V study cohort, samples were collected at baseline, at the end of treatment (week 8), and at 3-month follow-up.

Cohort 2 consists of 23 non-cirrhotic patients with chronic HCV infection recruited at the MHH outpatient clinic. Inclusion criteria included sustained virologic response (SVR) achieved with direct-acting antiviral (DAA) therapy, regular follow-up, and liver elastography < 14.5 kPa. Comprehensive exclusion criteria are listed in Supplementary Fig. S1. Blood sampling was performed at DAA therapy start (defined as baseline), end of treatment (week 8) and at follow-up visits 6 and 24 months after the start of therapy.

Cohort 3 included a cohort of 31 individuals with chronic hepatitis C and cirrhosis who achieved SVR after DAA treatment (Table 1). Samples were obtained at DAA therapy start (defined as baseline), after completion of treatment (week 8) and during a median long-term follow-up period of 24 months after therapy. A comprehensive description of baseline characteristics is provided in Table 1 and exclusion criteria are shown in supplementary Fig. S1.

Two SARS-CoV-2 cohorts, consisting of 142 patients recruited at the Long-COVID outpatient clinical MHH (project within the German Center for Lung Research (DZL)), were enrolled in the study. Significant immunosuppressive conditions (transplantation) or autoimmune diseases were excluded. Inclusion criteria included regular follow-up, confirmed positive PCR results between February 2020 and March 2021 and survival status (Table 2, 3 and 4). We distinguished the SARS-CoV-2 patients in two cohorts. Cohort one is including 103 patients as a cross-sectional cohort at three follow-ups. Cohort two is representing a longitudinal cohort including the acute infection (defined as date of positive PCR test) and four different follow ups. The study established the first positive PCR test as baseline, and subsequent assessments occurred at intervals up to 3 months, 6 months, 9 months, and 14 months after baseline. The severity of SARS-CoV-2 infection was characterized using the WHO Clinical Improvement Ordinal Scale (Table S1). The baseline characteristics for both SARS-CoV-2 cohorts are shown in Table 2, and the inclusion criteria are shown in Supplementary Fig. S2.

**Table 2:** SARS-CoV-2 Cohorts 1 and 2.

Characteristics	SARS-CoV-2 Cohort 1		SARS-CoV-2 Cohort 2	
Number of patients	Total	103	Total	39
Sex	Female	56	Female	18
	Male	47	Male	21
Mean age (range)		47.6 (19–81)		50.2 (18–83)
Mean BMI (SEM)		26.7 ± 4.9		26.6 ± 4.7
Severity (acute phase)	Mild	74	Mild	16
	Severe	15	Severe	7
	ICU	14	ICU	16
Number of patients at given time point	Acute phase			20
	3 months	36	3 months	16
	6 months	42	6 months	33
	9 months	25	9 months	38
			14 months	22

**Table 3:** SARS-CoV-2 Cohort 1.

Characteristics	Up to 3 months		Up to 6 months		Up to 9 months	
Number of patients		36		42		25
Sex	Female	18	Female	22	Female	16
	Male	18	Male	20	Male	9
Mean age (range)		44.8 (22–71)		50.4 (19–81)		46.8 (22–79)
Mean BMI (SEM)		25.2 ± 3.9		27.9 ± 5.2		27.1 ± 5.1
Severity in acute phase	Mild	25	Mild	27	Mild	22
	Severe	8	Severe	5	Severe	2
	ICU	3	ICU	10	ICU	1

**Table 4:** SARS-CoV-2 cohort 2.

Characteristics	ICU			Severe			Mild		
Number of patients	16			7			16		
Median age (range)	47.25 (18–74)			63.29 (47–83)			47.75 (23–72)		
Sex (female/male)	3/13			3/4			12/4		
Median BMI (range)	29.26 (19.7–42.6)			27.28 (23.2–30.5)			24.11 (17.4–29.7)		
Comorbidities	Lung disease		2	Lung disease		0	Lung disease		1
	Heart disease		3	Heart disease		3	Heart disease		0
	Diabetes mellitus		4	Diabetes mellitus		1	Diabetes mellitus		0
	Adiposities		6	Adiposities		1	Adiposities		0
Immunosuppression	1			0			0		
Glucocorticoids	3			0			0		
Remdesivir	7			0			0		
Smoker status	Never	11		Never	4		Never	5	
	Former	5		Former	3		Former	10	
	Active	0		Active	0		Active	0	
Fatigue status	T2	No	4	T2	No	0	T2	No	2
		Mild	3		Mild	2		Mild	0
		Extreme	2		Extreme	2		Extreme	1
	T3	No	6	T3	No	2	T3	No	6
		Mild	6		Mild	3		Mild	7
		Extreme	0		Extreme	1		Extreme	2
	T4	No	9	T4	No	4	T4	No	7
		Mild	5		Mild	2		Mild	7
		Extreme	1		Extreme	1		Extreme	2
	T5	No	5	T5	No	3	T5	No	1
		Mild	3		Mild	1		Mild	4
		Extreme	3		Extreme	0		Extreme	2
Median FEV1% (range)	T2	88.9 (68–107)		T2	105.8 (96–114)		T2	98 (92–103)	
	T3	87.6 (55–105)		T3	103.5 (94–110)		T3	98.9 (78–122)	
	T4	92.3 (51–131)		T4	102.4 (86–116)		T4	97.8 (69–123)	
	T5	86.3 (58–106)		T5	89 (79–99)		T5	94.9 (79–115)	

In addition, 30 healthy donors were included as controls (Table 5). Notably, two individuals with hypothyroidism and one individual with drug-requiring allergies were excluded.

**Table 5:** Healthy donor baseline characteristics.

Characteristics	Healthy donor cohort	
Number of patients	Total	30
Sex	Female	14
	Male	16
Mean age (range)		41 (25–65)
BMI		n.a.

Blood sampling time points for the cohorts were standardized to baseline (T1), marking the start of therapy in the HCV cohorts and the first positive PCR result for SARS-CoV-2 patients. Subsequent time points are defined as follows: T2 (2–3 months after baseline, corresponding to the end of 6–12 weeks of DAA therapy in HCV patients), T3 (5–6 months after baseline, corresponding to 12 weeks post-DAA therapy in HCV patients), T4 (8–9 months after baseline, representing 24 weeks post-DAA therapy in HCV patients), and T5 (long-term follow-up, up to 14 months after baseline). SARS-CoV-2 cohort 1 includes 103 cross-sectional patients, categorized by time point after first positive PCR test results. The second cohort includes 39 patients in a longitudinal follow up over all five selected time points.

The distinctive feature of all cohorts is the timing of the sample collection dates. Healthy donor patients sampling was conducted between October and December 2019, prior to the emergence of SARS-CoV-2 in Europe. Sample collection for the cohort of chronically infected HCV patients without cirrhosis began in July 2015 and ended in April 2018. For the SARS-CoV-2 patients, sample collection commenced in March 2020 and continued until August 2021. The majority of the samples were collected before the start of the vaccination campaign in Germany, ensuring the follow ups were not affected by the vaccination. A timeline was created to visually represent the sample collection periods in relation to the onset of vaccinations and the vaccine availability to people of the age 60 and younger (Fig. S3). All samples were collected according to the specific standard operating procedures of the Hannover Unified Biobank.

### Ethical approval

This article does not contain any studies with animals performed by any of the authors. The study protocol conformed to the ethical guidelines of the Declaration of Helsinki and the ethics committee of Hannover Medical School approved this study a priori (No. 9001_BO_K, No. 9472_BO_K_2020, broad consent: No. 2923-2015). Informed consent was obtained from all individual participants included in the study.

### SIM assay

The Quanterix HD SP-X Imaging and Analysis System™ was used to measure the plasma samples. The following panels were used in this study: Human Corplex cytokine panel 1 10-Plex array included IL-12p70 (interleukin 12 active heterodimer p70), IL-1β (interleukin-1 beta), IL-4 (interleukin-4), IL-5 (interleukin-5), IL-6 (interleukin-6), IL-8 (interleukin-8), TNFα (tumor necrosis factor alpha), IFNγ (interferon gamma), IL-10 (interleukine-10), IL-22 (interleukine-22). Simoa chemokine panel 1 4-plex kit contained four chemokines: IP-10 (interferon-gamma induced protein 10 kD; CXCL10), MCP1 (monocyte chemoattractant protein 1; CCL2), MIP1-β (Macrophage inflammatory protein 3-beta; CCL19), and ITAC (Interferon-inducible T-cell alpha chemoattractant; CXCL11). IL-4 and IL-5 were excluded from further analysis, due to being below the limit of detection. All plasma samples were processed according to standard biobanking protocols and stored at a minimum temperature of – 80 °C. For the experiments the samples were randomized and measured according to the manufacturer’s manual.

### Statistical analysis

For the statistical analysis of the SIM comparisons a Kruskal–Wallis with Dunn’s correction was employed using GraphPad Prism version 9.0. Pearson correlations were calculated to detect associations between clinical outcome and the SIM measurements. P-values for the correlation analysis were adjusted using the Benjamini–Hochberg method.

### Linear models for temporal and cross-cohort analysis

All the SIM measurements were log2 transformed. For the different cohorts and the three time points evaluated (T1, T2, T3), linear models were fitted for each SIM individually, where the relationship between SIM expression and time was evaluated using age, sex and cohort as covariates. Linear models were fitted for each SIM from each cohort, taking time as a continuous variable, resulting in an estimate value for the SIM of each cohort. The estimate explains the linear relationship of SIM molecule against time for each cohort. Therefore, a positive estimate represents a positive relationship of the SIM (upregulation) with time, while a negative estimate represents a negative relationship. Furthermore, the value of estimate describes the strength of this relationship. The density describes the distribution of all SIM estimates (from linear models) for each of the cohort. Linear models also included age and sex as co-variates. Time was taken as a continuous covariate and a density plot estimate for time variable for each cohort was generated. For the cross-cohort comparison at each time-point, each HCV cohort was evaluated using SARS-CoV-2 Cohort 2 samples as a baseline. Density plot estimates were generated for each time point for the three HCV cohorts, compared against SARS-CoV-2 Cohort 2. Linear models were fitted for each time point (categorical variable) to compare the SIM expression between SARS-CoV-2 cohort 2 and either of the three HCV cohorts. In this analysis, the estimate describes the expression of SIM in HCV cohorts with expression of SIM in SARS-CoV-2 cohort 2 as baseline measurement. Therefore, the density describes the distribution of estimate of all SIMs in each of the three HCV cohorts compared to SARS-CoV-2 cohort 2. Linear models also included age and sex as co-variates.

## Results

### Temporal dynamics of SIM in viral infections

**Fig. 1:**
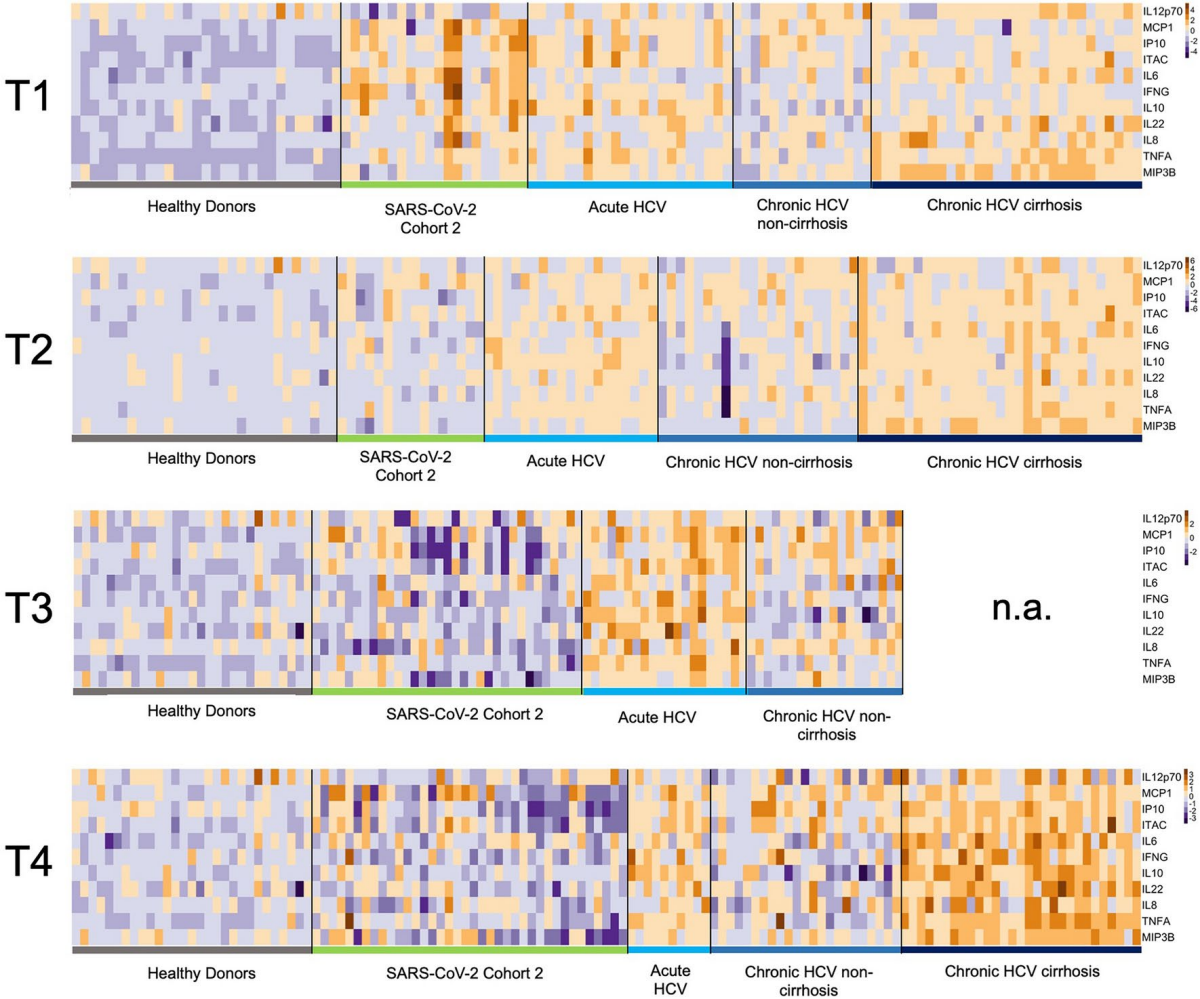
Soluble inflammatory mediators (SIM) over time in SARS-CoV-2 and HCV cohorts. A logarithmic scale heatmap illustrating the concentrations of individual cytokines and chemokines relative to the corresponding median concentrations at different time points (T1-4). Dark blue represents decreased SIM concentrations, while dark brown indicates increased SIM concentrations. Healthy donor levels at T1 are maintained across all time points. T1 corresponds to baseline (start of therapy/positive PCR result), T2 = 3 months, T3 = 6 months, and T4 = 9 months. Notably, data for the long-term follow-up (T5) are presented for the chronic HCV cohort with cirrhosis, as T4 data is missing.

We employed the Quanterix HD SP-X Imaging and Analysis System™ to assess the impact of different viral infections and the dynamics of soluble inflammatory mediators (SIM). A heatmap was created to visualize the measured concentrations at various time points (Fig. 1). At T1, baseline assessments for the HCV and SARS-CoV-2 cohorts showed elevated SIM activity (IL-6 and IL-22) compared to healthy donors. Following viral clearance at T2, all cohorts exhibited a reduction in SIM activity, indicating a decline in the immune response.

At T3 (six months), the SARS-CoV-2 cohort showed decreased SIM activity, particularly for MCP1, IP-10, and ITAC, compared to the median values of the selected SIMs. In contrast, the acute HCV and non-cirrhotic HCV cohorts displayed higher SIM responses than the control group and the SARS-CoV-2 cohort post-viral clearance. Notably, cirrhotic patients exhibited the highest SIM activity during long-term follow-up.

At T4 (nine months), SIM activity in the SARS-CoV-2 cohort remained decreased. The acute HCV and chronic non-cirrhotic HCV cohorts demonstrated a generalized decline in SIM activity, distinguishing them from the cirrhotic HCV group.

### Comparative analysis of SIM dynamics in viral infections

**Fig. 2:**
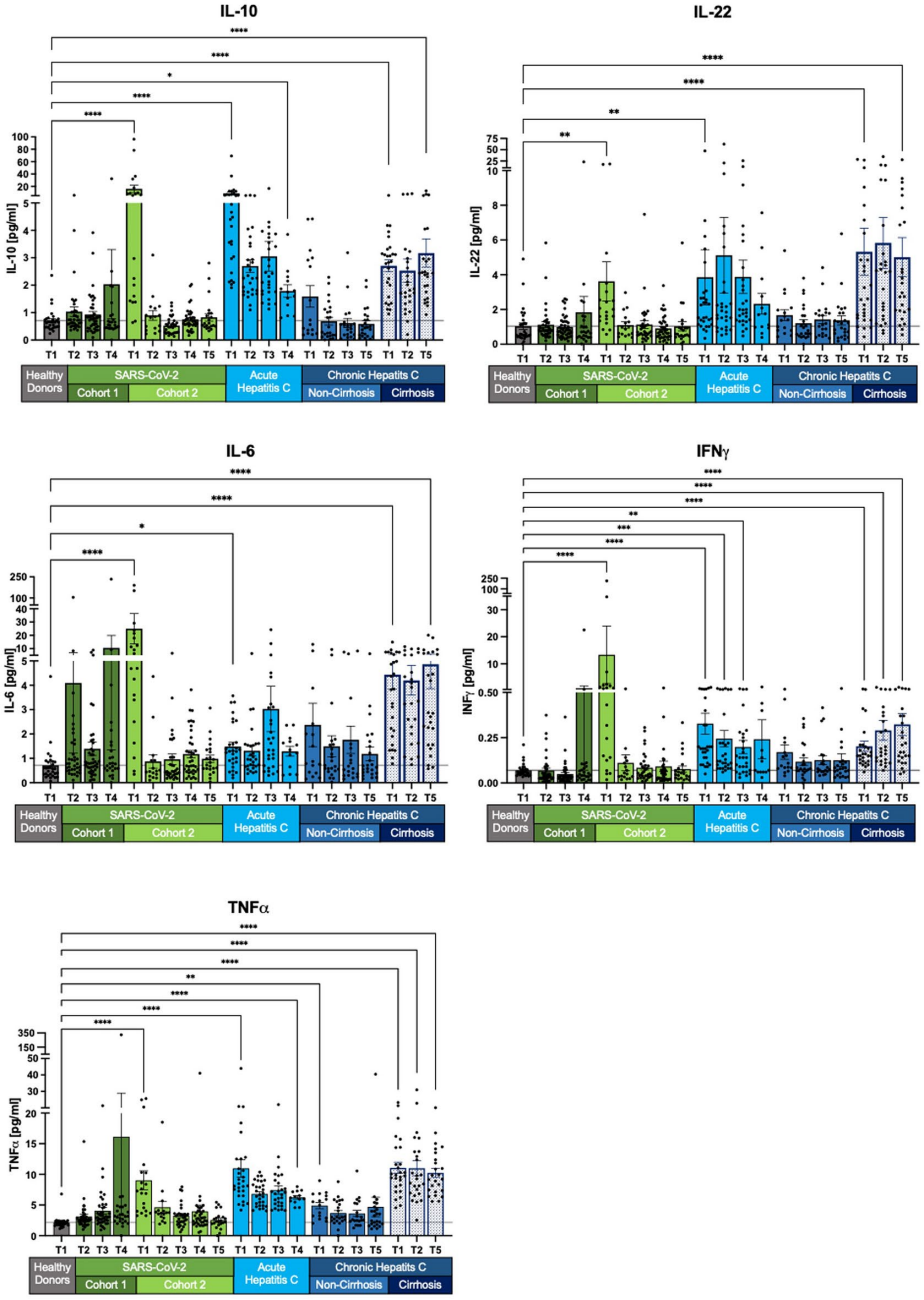
Decline of selected SIM levels across all cohorts except chronic HCV with cirrhosis. IL-10, IL-22, TNFα, IFNγ, and IL-6 displayed normalization in SARS-CoV-2 cohort 2. Acute HCV patients exhibited overall increased SIM levels. Non-cirrhotic HCV patients returned to healthy levels after achieving sustained virologic response (SVR). However, chronic cirrhotic HCV patients showed persistent SIM levels with no significant change compared to T1. Measurements are presented as the mean with standard error. The Kruskal–Wallis test with Dunn’s correction was applied for comparisons between healthy donors and the different cohorts at T1 and the last follow-up. Two-tailed p-values were considered significant at < 0.05 (*), < 0.01 (**), < 0.001 (***) and < 0.0001 (****).

**Fig. 3:**
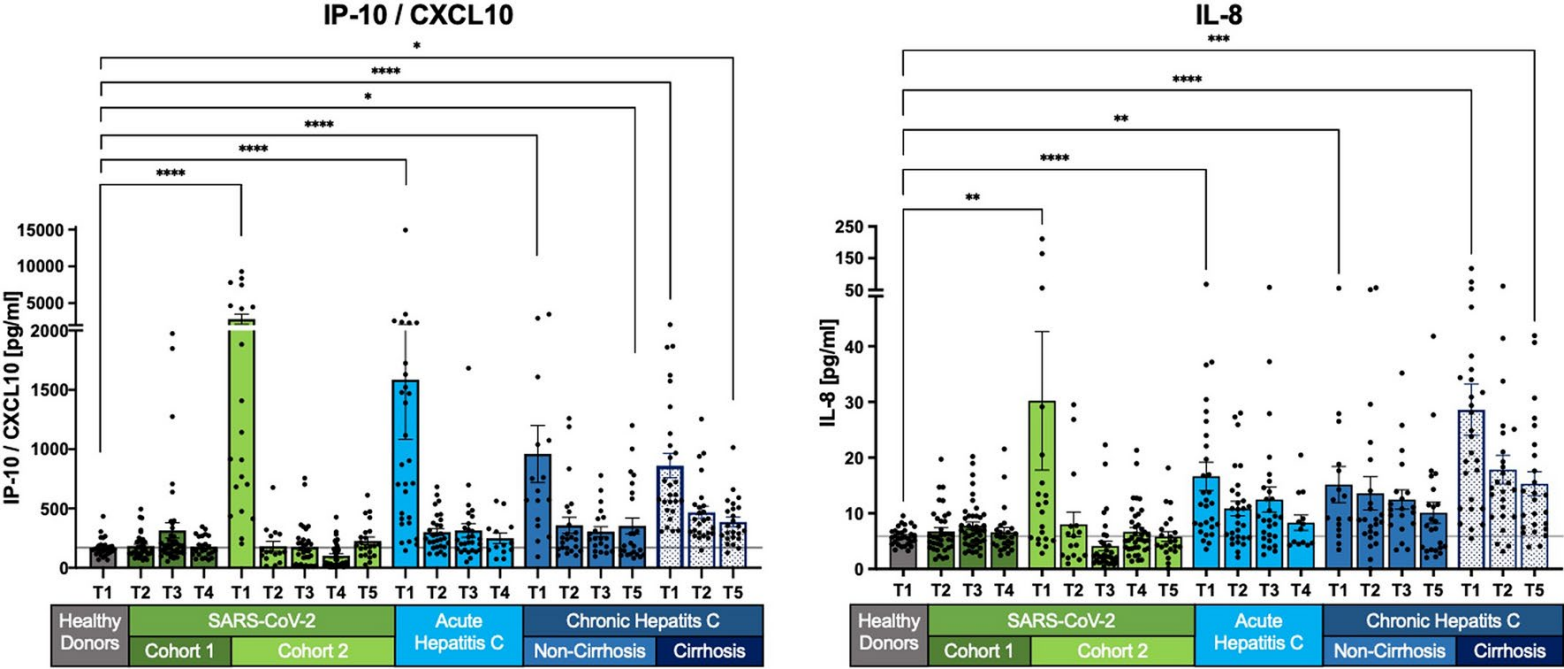
Normalization of selected SIM levels across most cohorts, persistent elevation in chronic HCV with cirrhosis. IL-8 and IP-10/CXCL10 levels normalize to healthy levels in SARS-CoV-2, acute HCV, and non-cirrhotic HCV patients. However, chronic HCV patients with cirrhosis show an initial decrease, followed by persistent elevation, without full recovery. Measurements are shown as the mean with the standard error of the mean. The Kruskal–Wallis test with Dunn’s correction was applied to compare healthy donors and the different cohorts at T1 and the last follow-up. Two-tailed P values < 0.05 (*), < 0.01 (**), < 0.001 (***) and < 0.0001 (****) were considered significant.

Further insights into the dynamics of individual SIMs are presented in Fig. 2, 3, 4. We observed a trend across most SIMs, with a significant increase in concentrations during acute SARS-CoV-2 infections compared to acute and chronic HCV infections. Notably, over time, the SIM levels in SARS-CoV-2 patients predominantly revert to those of healthy donors.

**Fig. 4:**
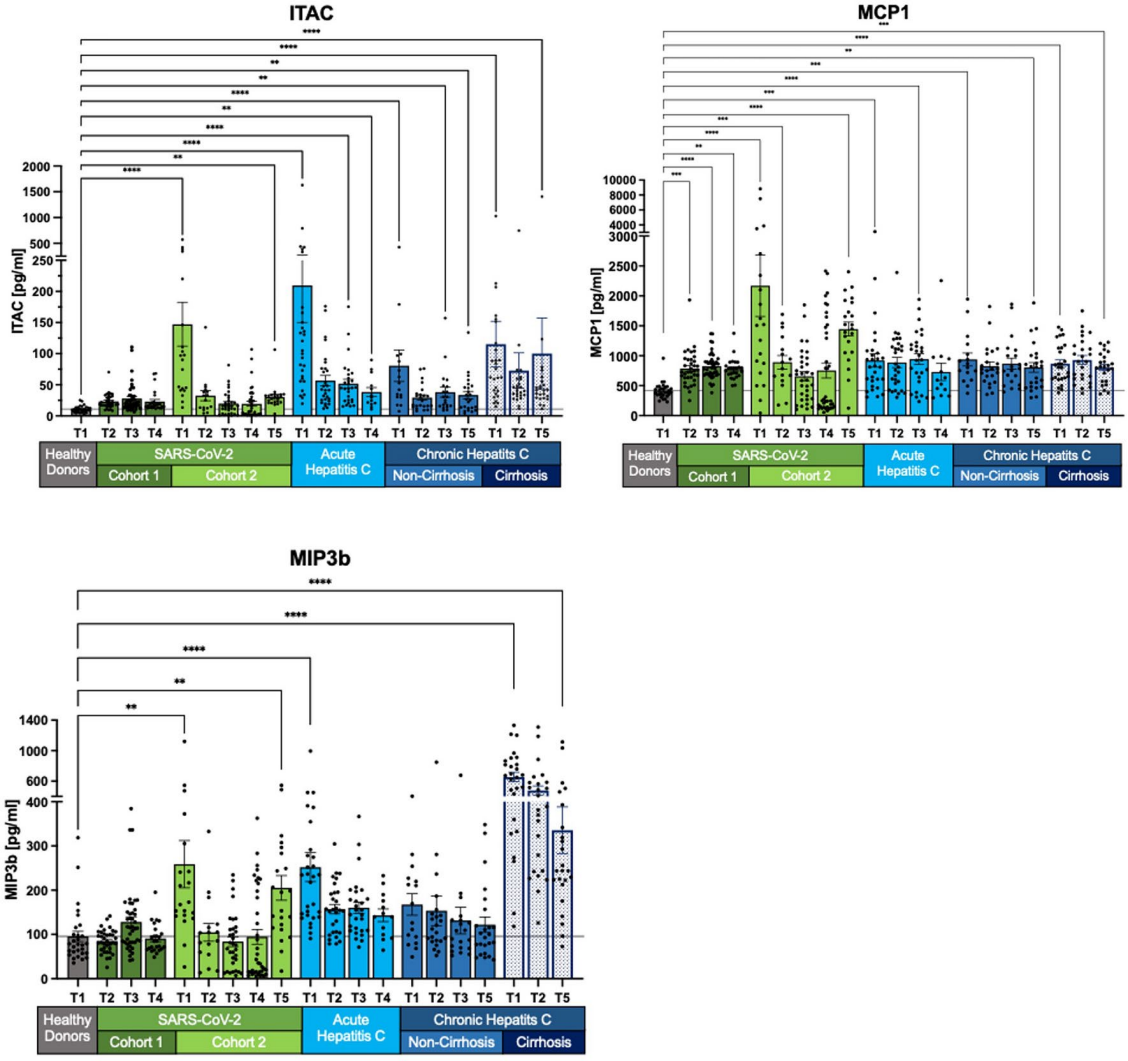
Prolonged elevation of chemokines MCP1, ITAC, and MIP-3β in SARS-CoV-2 and HCV patients. In SARS-CoV-2 cohort 2, MCP1, ITAC and MIP-3β levels initially decline, before increasing again. In HCV patients, SIM levels decrease compared to healthy controls but remain elevated, with significant discrepancies in concentration levels depending on the stage of HCV infection. Measurements are shown as mean with its standard error. Kruskal–Wallis with Dunn’s correction has been used for the healthy donors and the different cohorts at T1 and the last follow-up. Two-tailed P values < 0.05 (*), < 0.01 (**), < 0.001 (***) and < 0.0001 (****) were considered significant.

IL-10, IL-22, TNFα, IFNγ, and IL-6 are elevated during SARS-CoV-2 infection and return to baseline levels after viral clearance from follow-up T2 to T5 (Fig. 2). However, in the cross-sectional cohort 1 of SARS-CoV-2, patients exhibit increased mean values at T4, despite individual SIM concentrations mostly aligning with healthy donor levels. The elevated mean values can be linked to outliers. Conversely, patients with acute and chronic HCV infections display different patterns. In non-cirrhotic patients with chronic HCV, SIM levels normalize to near-healthy levels, while cirrhotic patients maintain higher SIM expression. These values exceed those of healthy controls and also remain at the long-term follow-up (T5) after virus elimination (all p < 0.0001) (Fig. 2).

IL-8 and CXCL-10 (IP-10) exhibit elevated levels at baseline (T1) but show no statistically significant difference from healthy levels at T2 (after 3 months/SVR) in SARS-CoV-2, acute HCV, and chronic non-cirrhotic HCV patients (Fig. 3). Persistent alterations are observed only in chronic HCV patients with cirrhosis, displaying a distinct long-term pattern compared to IL-10, IL-22, TNFα, IFNγ, and IL-6 (Fig. 2). A notable decrease in concentration levels from T1 to T5, including IP-10/CXCL10 (T1: p < 0.0001; T5: p < 0.05) and IL-8 (T1: p < 0.0001; T5 p < 0.001), is noticeable (Fig. 3).

### Dynamics of chemokines MCP1, ITAC and MIP-3β with prolonged elevation in SARS-CoV-2 and HCV

MCP1, ITAC, and MIP-3β concentrations exhibit dynamic changes, with SARS-CoV-2 cohort 2 showing an initial decline followed by a long-term increase. MIP-3β levels, in particular, significantly increase at follow-up T5 compared to T4, reaching almost 80% of baseline T1 levels. MCP1 levels show no long-term recovery, returning to T1 levels by T5 in SARS-CoV-2 cohort 2 (T1: p < 0.0001; T5: p < 0.0001). Similarly, ITAC concentrations in the long-term follow-up at T5 are elevated in SARS-CoV-2 cohort 2 (T1: p < 0.0001; T5: p < 0.01).

For all HCV cohorts, there is a general decrease in concentrations, although levels remain above those of healthy controls. Acute HCV patients exhibit the most significant decline in MIP-3β levels (T1: p < 0.0001; T4: p > 0.05). Chronic non-cirrhotic HCV patients also show a decrease in MIP-3β concentrations; however, this change is not statistically significant when compared to healthy donor levels. In contrast, chronic cirrhotic HCV patients have highly significant MIP-3β levels compared to healthy donors, indicating that their decrease at T5 is not reflected in the p-values.

ITAC concentrations decline in both acute HCV and chronic non-cirrhotic HCV patients at T4 and T5 (both p < 0.01). Chronic cirrhotic HCV patients also exhibit a decrease in ITAC levels, although an outlier at 1406 pg/ml skews the mean value. Other concentrations ranged from 11.57 pg/ml to 123 pg/ml.

MCP1 levels remain elevated in both acute and chronic HCV patients, irrespective of cirrhosis. Despite small but significant changes, these levels are higher than those of healthy donors. For example, in chronic non-cirrhotic HCV patients, MCP1 levels change from p < 0.001 to p < 0.01 (Fig. 4).

### Comparative analysis of SIM dynamics using linear models

**Fig. 5:**
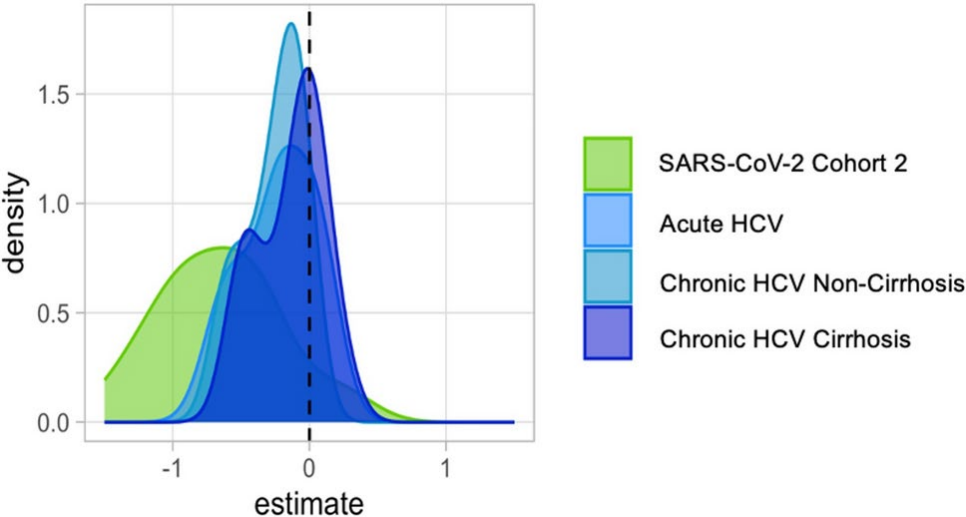
Time-adjusted distribution of Log2-transformed SIM estimates across cohorts, evaluated with age and sex as covariates. Distribution of estimate for the SIM evaluated for each cohort per time. All the SIM measurements were log2 transformed. For each cohort, linear models were fitted for each SIM, where SIM expression was evaluated with age, sex, and time as covariates. Time was taken as a continuous covariate. A density plot estimate for time variable for each cohort was plotted.

We employed linear models to evaluate changes in SIM expression over time for each cohort. A negative estimate indicated a decrease in SIM expression, while a positive estimate suggested either an increase or stable expression. For almost all SIMs, the SARS-CoV-2 cohort 2 exhibited decreased density, while most SIMs in the HCV cohorts also showed a negative association over time (Fig. 5).

**Fig. 6:**
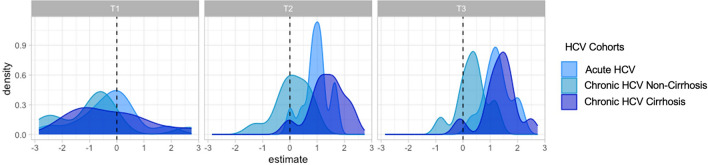
Density plots of Log2-transformed SIM estimates at T1, T2, and T3 across HCV cohorts compared to SARS-CoV-2 Cohort 2 baseline. Distribution of estimate for SIM for each time point evaluated across cohorts, with SARS-CoV-2 cohort 2 as baseline cohort. All the SIM measurements were log2 transformed. For each of the three-time points evaluated (T1, T2, T3), linear models were fitted for each SIM, where SIM expression was evaluated with age, sex and cohort as covariates. Each HCV cohort for each time point was evaluated, with SARS-CoV-2 cohort 2 samples were taken as a baseline for comparison. A density plot estimate was plotted for each time point and for the three HCV cohorts evaluated against the SARS-CoV-2 cohort 2.

Additionally, we fitted linear models for each time point to assess changing SIM expression in HCV cohorts compared to the SARS-CoV-2 cohort 2. At T1, SIM expression in all HCV cohorts mirrored that of the SARS-CoV-2 cohort. However, at T2 and T3, almost all SIMs in the HCV cohorts showed a positive association compared to SARS-CoV-2 cohort 2, indicating higher levels in HCV patients at these time points (Fig. 6).

### Association analysis of SIM and fatigue in SARS-CoV-2 cohorts

All SARS-CoV-2 patients underwent comprehensive fatigue screening and longitudinal evaluation (Supplementary Fig. S4). To identify associations between fatigue syndrome and the systemic inflammatory milieu (SIM), we performed a correlation analysis. Interestingly, none of the analyzed SIMs were directly associated with the fatigue scores collected during follow-up (p.adj > 0.05).

### Comparative analysis of SARS-CoV-2 cohort 2 and chronic HCV non-cirrhosis cohort for sex, age and BMI

In order to identify the impact of the variables sex, age and BMI, we compared them in selected SIMs (IL-6, IP-10, ITAC and MIP3β) as shown in supplementary Fig. S5. Mann–Whitney-Test has been used the distribution of the variables (sex, age and BMI) within one timepoint. Two-tailed P values < 0.05 (*), < 0.01 (**), < 0.001 (***) and < 0.0001 (****) were considered significant. P-values were adjusted using the Benjamini–Hochberg method and a false discovery rate of p = 0.05. Nnone of the variables show a significantly difference (p.adj > 0.05).

### Comparative analysis of SARS-CoV-2 cohort 2 for steroidal and/or antiviral therapy during the acute phase of infection

Supplementary Fig. S6  shows the differences between steroids and/or antiviral therapy during the acute phase of infection in selected SIMs (IL-6, IP-10, ITAC and MIP3β). Mann–Whitney-Test has been used the distribution of the variables (sex, age and BMI) within one timepoint. Two-tailed P values < 0.05 (*), < 0.01 (**), < 0.001 (***) and < 0.0001 (****) were considered significant. P-values were adjusted using the Benjamini–Hochberg method and a false discovery rate of p = 0.05. None of the variables show a significantly difference (p.adj > 0.05).

## Discussion

Our comprehensive investigation of SIM dynamics in COVID-19 and HCV has provided valuable insights into immune responses after viral infections. Our results reveal different temporal patterns of cytokine and chemokine concentrations in response to SARS-CoV-2 and HCV infections and shed light on potential differences in immune modulation and recovery trajectories.

An interesting observation from our analysis is the more rapid decline in SIM expression after SARS-CoV-2 infection compared with all HCV cohorts. The rapid decline in SIM level in SARS-CoV-2 patients at month 3 after recovery suggests more efficient modulation of the immune response and is indicative for rapid recovery in most individuals. This may indicate that SARS-CoV-2 induces a robust yet controlled immune reaction that subsides more promptly than in HCV infections. The distinct SIM dynamics observed in response to these two viral infections underscore the complex interplay between viral pathogens and the host immune system.

The observed differences in SIM dynamics between SARS-CoV-2 and HCV cohorts may also be attributed to the nature of the infections themselves. Long-term chronic exposure to HCV in the chronic cohorts can certainly explain the slower decline in SIM after HCV elimination by antiviral therapy. However, we also observed comparable data at follow-up up to 6 months in those after acute HCV infection. SARS-CoV-2 primarily infects the respiratory tract, resulting in acute respiratory symptoms, whereas HCV primarily infects the liver via the bloodstream. The different anatomical sites of infection could influence the systemic immune response and the kinetics of SIM expression. The liver, as an immunological organ, has a distinct ability to induce antigen-specific tolerance and modulate immune responses^25,26^, possibly leading to prolonged dysregulation of the immune system after infection. Importantly, steroids administered in the early phase of acute SARS-CoV-2 infection may also affect SIM dynamics post-infection. However, the SARS-CoV-2 longitudinal cohort sample started before steroids became standard treatment, and we found no obvious differences in the few patients who received steroids. However, the effects of steroids as well as antiviral therapy during acute SAR-CoV-2 infection on long-term outcomes need to be further investigated.

The effects of cirrhosis in HCV infection have been shown to be an important factor in pronounced immune modulation. The higher SIM activity observed in cirrhotic HCV patients even after viral clearance is consistent with the notion that liver cirrhosis can perpetuate immune dysregulation^27^. This phenomenon likely contributes to the increased and persistent changes in SIM concentrations observed in this cohort and warrants further in-depth investigation. For example, it would be useful to investigate whether certain inflammatory responses correlate with the risk of developing hepatocellular carcinoma after viral elimination.

Comparison of SIM expression between HCV and Long-COVID cohorts at different time points provides additional insights and may help explain clinical observations. For instance, research indicates that individuals recovering from SARS-CoV-2 infection exhibited increased resting heart rates specifically during the initial 11 weeks post-infection, as evidenced by digital data from participants of the German Corona Data Donation Project^28^. This aligns roughly with the 3-month period after which SIM levels tend to normalize. Another study analyzing clinical data collected at a dedicated post-COVID 19 consultation at a rehabilitation outpatient clinic showed significant, albeit slow regression over a 6-month follow-up period^29^. Consistent with our results, some of the individuals still had elevated SIM levels after 6–9 months compared with healthy controls, e.g., IL-6, which has been attributed to long-COVID in several studies^30^. In addition, our data are coherent with a multi-omics study indicating that the immune system of convalescent COVID-19 patients largely returns to a homeostatic level similar to that of healthy individuals, despite minor persistent differences observed at the transcriptomic level in monocytes^31^. This may explain the increase in ITAC (CXCL11), MCP1 (CCL2) and MIP3b (CCL19) in individuals with long COVID-19 in our study, as these SIM are mainly expressed by monocytes, macrophages or dendritic cells. However, this rebound may also be influenced by vaccine responses^32^, given that vaccination started during the last period of the sampling. In addition, we cannot completely rule out new asymptomatic SARS-CoV-2 infections between these visits.

When analyzing patients after SARS-CoV-2 infection, we need to discuss possible biases. For example, individuals attending a specialized post-COVID rehabilitation outpatient clinic for neurocognitive problems might have different symptoms and SIM profiles than individuals attending the clinic for respiratory symptoms, as it was the case in our cohort. And indeed, a recent meta-analysis examining biomarkers in long-COVID revealed different SIM patterns in individuals with neurologic symptoms (CCL2, IFN-γ, IL-4, IL-6, TNF-α) versus individuals with pulmonary symptoms (CXCL-10, IFN-β, IL-1α, IL-6, TGF-β)^30^. In our longitudinal cohort, we did not detect a direct association between a specific SIM pattern and clinical manifestations, including fatigue. Here, it is important to note that our longitudinal cohort included individuals who were followed up after severe COVID-19 disease and followed primarily because of the initially severe respiratory symptoms. In a broader context, the lasting effects of SARS-CoV-2 infection may not be as common as generally assumed^33^, but rather limited to a subset of individuals with specific long-COVID symptoms who seek treatment in specialized centers. To date, studies suggest that approximately 10% of SARS-CoV-2 infected individuals experience long-term effects^4^. Interestingly, our cross-sectional cohort, which consisted specifically of individuals who participated because of long-COVID symptoms, had higher levels of IL6, TNFα, IFNγ at month 9. This supports the notion that SIM may be related to long-COVID symptoms. Nevertheless, we encountered limitations in correlating specific SIM with clinical parameters. It may be important to study dynamics rather than absolute values. This highlights that the dynamics of SIM should be further studied in various but homogeneous post-COVID cohorts.

Limitations of our study encompass its retrospective design and the restricted sample size, especially concerning patients with severe long-COVID symptoms, notably neurological manifestations. In addition, we lacked an assessment of extrahepatic manifestations such as fatigue in HCV-infected patients, and it is worth noting that a proportion of these patients may still have relevant neurocognitive impairment after HCV elimination^14,15^, which still warrants correlation with the expression of soluble inflammatory markers. The study cohort is heterogenous and lacks sufficient size to analyse subgroups, e.g. the effects of sex, age and obesity. Analysing the effect of these factors would be of great interest as for example post COVID-19 sick-leave has been linked to female sex, obesity and age between 36 and 45 years^34^. Thus, our study should be interpreted as exploratory, warranting further validation in larger, more homogenous cohorts.

In summary, our study provides insights into the dynamics of systemic immune mediators following SARS-CoV-2 and HCV infections and reveals distinct patterns that contribute to our understanding of immune modulation and recovery trajectories in viral diseases. The interplay between viral pathogens, immune responses and underlying health conditions needs to be further explored to unravel the mechanisms underlying these differences and to investigate their impact on the long-term consequences of viral infections.

## Supplementary Information

Supplementary Information.

## Data Availability

The datasets generated during and analysed during the current study are available from the corresponding author on reasonable request.

## Abbreviations

ARDS: Acute respiratory distress syndrome
CFS: Chronic fatigue syndrome
ALT: Alanine aminotransferase
AST: Aspartate aminotransferase
DAA: Direct-acting antiviral
HCC: Hepatocellular carcinoma
HCV: Hepatitis C virus
SIM: Soluble immune mediators
SVR: Sustained virological response
