# Supplementary material for: Different dynamics of soluble inflammatory mediators after clearance of respiratory SARS-CoV-2 versus blood-borne hepatitis C virus infections
Source: Sci Rep. 2024 Nov 22;14:29013. doi: 10.1038/s41598-024-79909-8 (PMC11584618; doi:10.1038/s41598-024-79909-8)
Supplement: Supplementary file 1 — Supplementary Information. [file 41598_2024_79909_MOESM1_ESM.docx]

**Different dynamics of soluble inflammatory mediators after clearance of respiratory SARS-CoV-2 versus blood-borne hepatitis C virus infections**

Antonia Zeuzem^1,2,3,4,5^, Saumya Dileep Kumar ^2,4^ , Carlos Oltmanns ^1,2,3,4,5^ , Moana Witte^1,2,3,4,5^ , Jasmin Mischke ^1,2,3,4,5^ , Nora Drick ^6,7^ , Jan Fuge ^6,7^ , Isabell Pink ^6,7^ , Jan Tauwaldt^1,2,3,4,5^, Jennifer Debarry ^2,4^ , Thomas Illig ^8^ , Heiner Wedemeyer^1,3,5^, Benjamin Maasoumy^1^, Yang Li ^2,4,5,9^ , Anke R.M. Kraft^1,2,3,4,5^, Markus Cornberg^1,2,3,4,5^

^1^Department of Gastroenterology, Hepatology, Infectious Diseases and Endocrinology, Hannover Medical School, Carl-Neuberg-Straße 1, 30625 Hannover, Germany

^2^Centre for Individualised Infection Medicine (CiiM), a joint venture between Helmholtz-Centre for Infection Research and Hannover Medical School, Feodor-Lynen-Straße 11, 30625 Hannover, Germany

^3^German Center for Infection Research (DZIF), partner site Hannover-Braunschweig Germany

^4^TWINCORE, Centre of Experimental and Clinical Infection Research, a joint venture between Helmholtz-Centre for Infection Research and Hannover Medical School, Feodor-Lynen-Straße 7, 30625 Hannover, Germany

^5^Cluster of Excellence RESIST (EXC 2155), Hannover Medical School, Carl-Neuberg-Straße 1, 30625 Hannover, Germany

^6^ Biomedical Research in Endstage and Obstructive Lung Disease Hannover (BREATH), German Center for Lung Research (DZL), Hannover, Germany.

^7^ Department of Respiratory Medicine and Infectious Diseases, Hannover Medical School (MHH), Hannover, Germany

^8^ Hannover Unified Biobank (HUB), Hannover Medical School (MHH), Germany

^9^ Department of Internal Medicine and Radboud Institute for Molecular Life Sciences, Radboud University Medical Center, Nijmegen, the Netherlands

**Supplement**

**Table S1: Characterization of Severity Grade of a COVID-19**

| Severity | Description | Ordinal Scale of WHO Clinical Improvement |
| --- | --- | --- |
| No infection | No COVID-19 positive result | 0 |
| Mild | Ambulatory | 1-2 |
| Severe | Hospitalized | 3-4 |
| Severe/ICU | Intensive Care Unit | 5-7 |
| Deceased | Death | 8 |

**Figure S1: Selection criteria for chronic HCV cohorts**

799 patients with chronic HCV monoinfection treated with direct-acting antivirals between 01/2014 and 11/2019

500 patients with samples available at therapy start

Patients without written consent or available samples at therapy start

31 chronic HCV patients with cirrhosis

23 chronic HCV non-cirrhosis patients

Fibroscan > 14.5 kPA

eGFR < 30ml/min

Liver decompensation (HE, Ascites)

DAA relapse

>1 Interferon therapy attempt

Ribavarin treatment

**Figure S2: Selection criteria for SARS-CoV-2 cohorts**

269 patients in the outpatient clinic 09/2021

198 patients with outpatient clinical data and plasma samples

142 patients with clinical data, plasma samples and regular follow-up visits

Longitudinal cohort: 39 patients with ≥ 2 follow up

Cross-Sectional cohort: 103 patients with < 2 follow up visits

Unavailable samples at Hannover Unified Biobank (HUB): 71 patients excluded

Regular follow up visits similar to standard procedure to gastroenterology outpatient clinic: 56 patients excluded

**Figure S3. Sample Collection Timeline of SARS-CoV-2 Cohorts 1 and 2**

**Figure S4: Association analysis of inflammatory milieu and fatigue in SARS-CoV-2 cohorts**

**
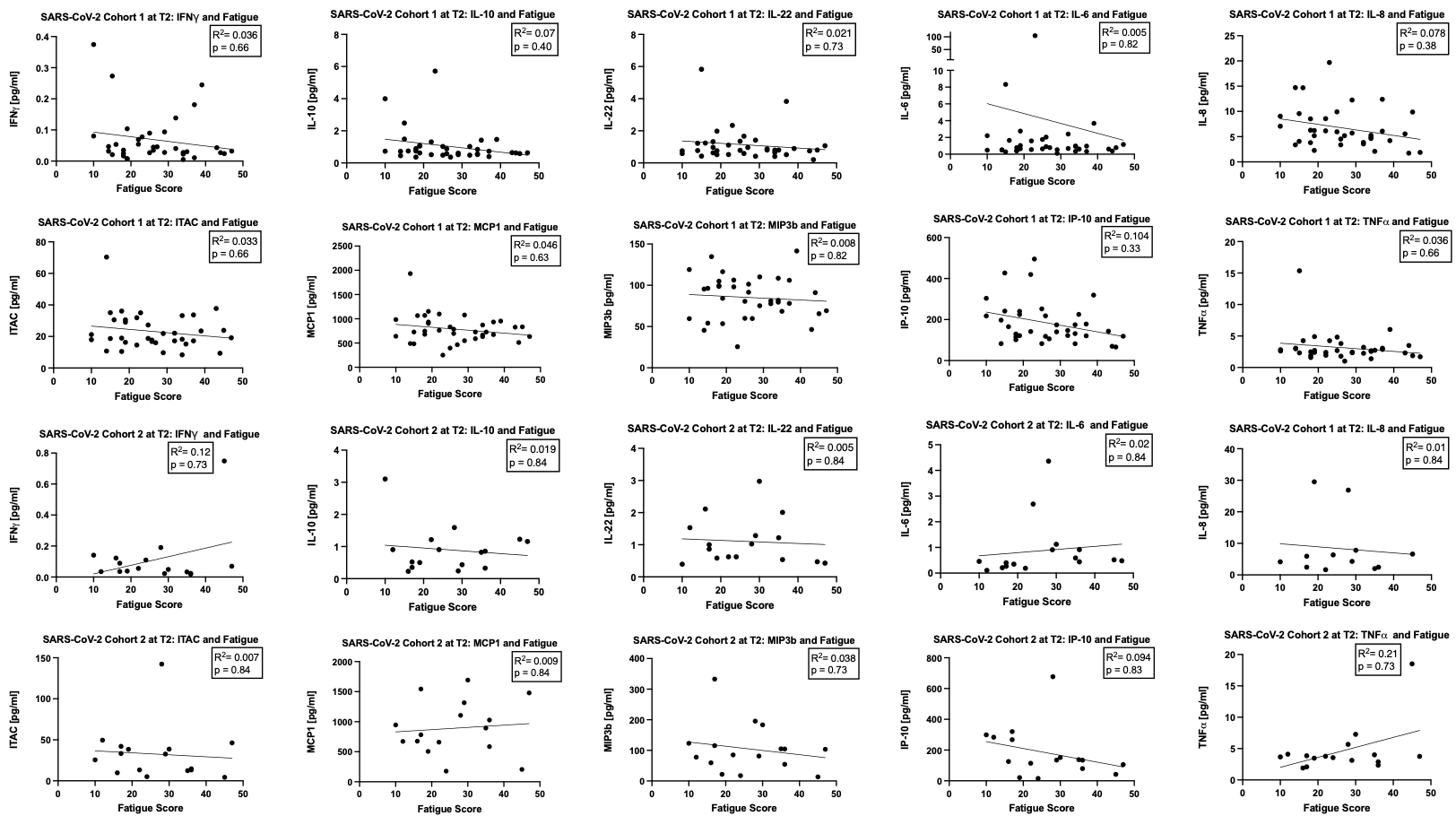
**

**
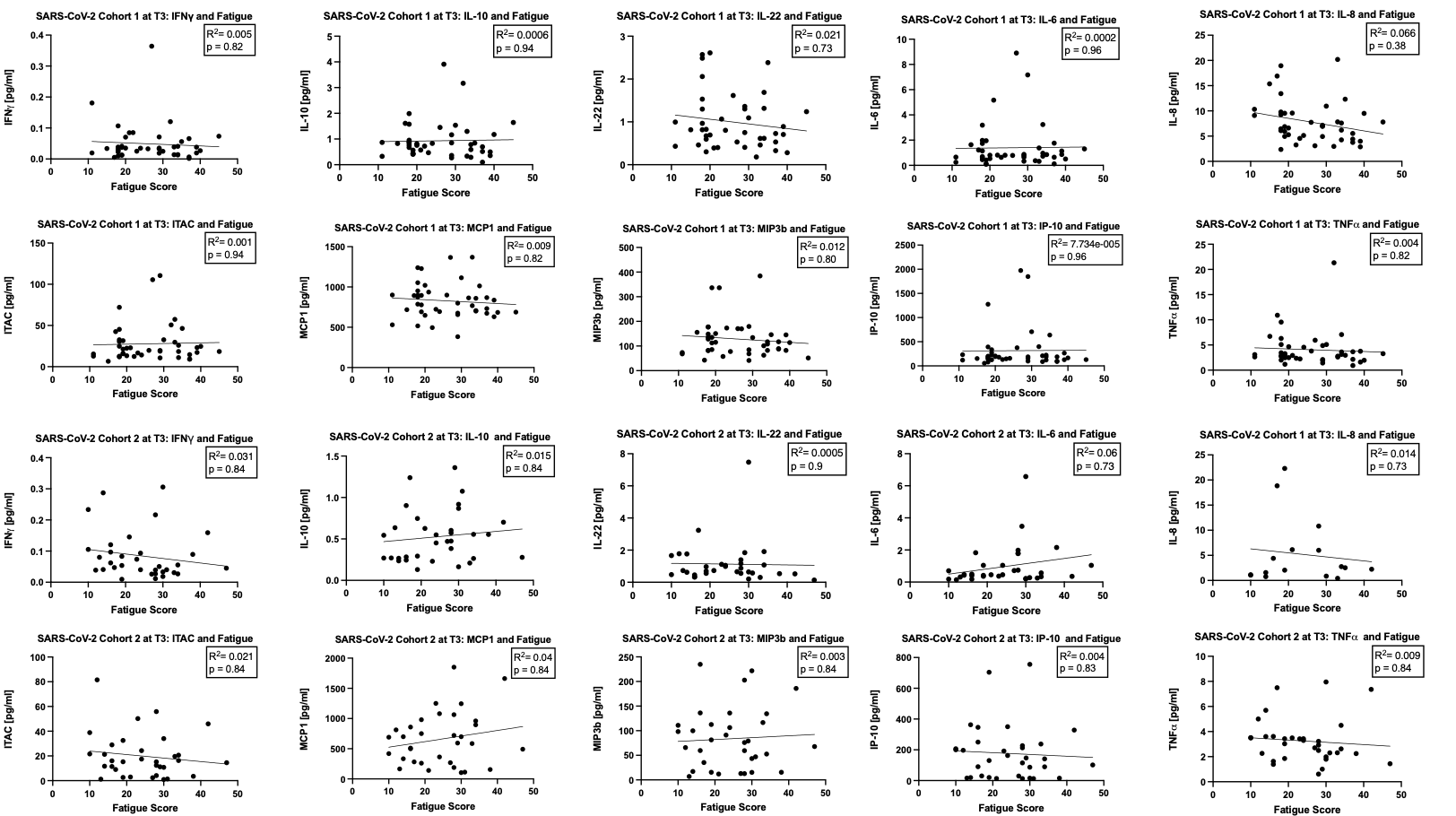
**

**
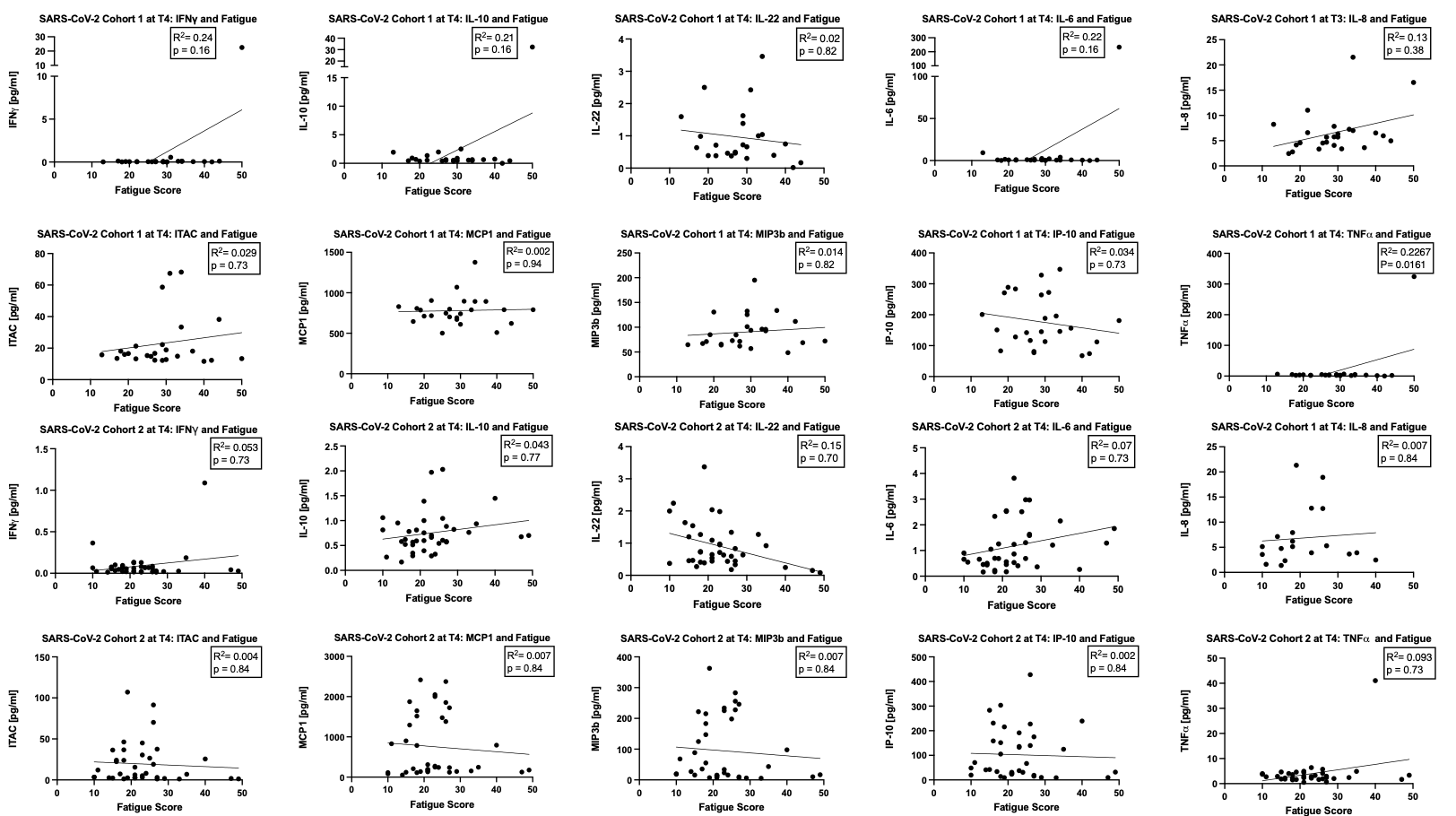
**

**
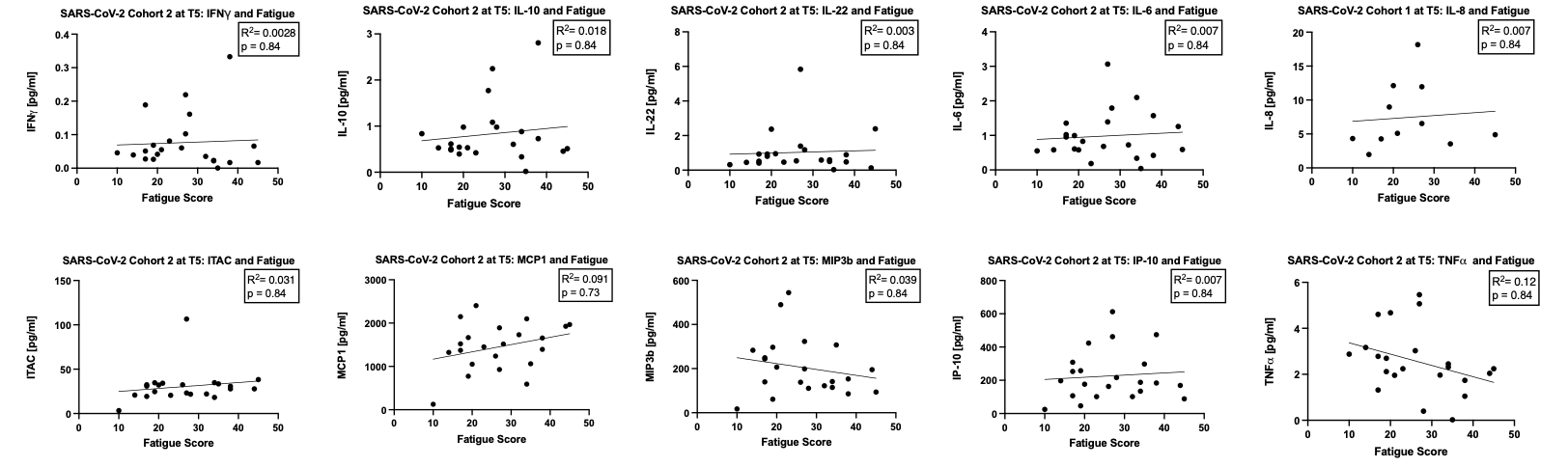
**

**Figure S5. Comparison analysis of SARS-CoV-2 cohort 2 and chronic HCV non-cirrhosis for sex, age and BMI**

**S6 Comparison analysis of SARS-CoV-2 cohort 2 for steroidal and/or antiviral therapy during the acute phase of infection**
